# Cognitive Decline in Adults Aged 65 and Older in Cumbayá, Quito, Ecuador: Prevalence and Risk Factors

**DOI:** 10.7759/cureus.3269

**Published:** 2018-09-07

**Authors:** Patricio H Espinosa del Pozo, Patricio S Espinosa, Eduardo A Donadi, Edson Z Martinez, Juan C Salazar-Uribe, Marco A Guerrero, Julio C Moriguti, Mishell C Colcha, Susana E Garcia, Raquel Naranjo, Wilson E Altamirano, Adriana Y Koek

**Affiliations:** 1 Neurosciences, Universidad Central Del Ecuador, Quito, ECU; 2 Neurology, Marcus Neuroscience Institute, Boca Raton Regional Hospital, Boca Raton, USA; 3 Departamento De Clinica, Facultad De Medicina Ribeirão Preto, Universidad De São Paulo, Ribeirão Preto, BRA; 4 Social Medicine, Ribeirão Preto Medical School, University of São Paulo, Ribeirão Preto, BRA; 5 Statistics, Universidad Nacional De Colombia (national University of Colombia), Medellín, COL; 6 Neurosciences Unit, College of Medical Sciences, Central University of Ecuador, Quito, ECU; 7 Departamento De Geriatria, Facultad De Medicina Ribeirão Preto, Universidad De São Paulo, Ribeirão, BRA; 8 Unidad De Neurociencias, Facultad De Ciencias Médicas, Universidad Central Del Ecuador, Quito, ECU; 9 Clinical Biomedical Science, Florida Atlantic University Charles E. Schmidt College of Medicine, Boca Raton, USA

**Keywords:** dementia, cognitive impairment, cognitive decline, alzheimer disease, neurodegenerative disease, neuroepidemiology, ecuador

## Abstract

Objective

To assess the prevalence of and risk factors for cognitive decline and dementia in individuals greater than 65 years of age in Cumbayá, Quito, Ecuador.

Methods

This is a cross-sectional observational study that was carried out in adults over age 65. The Mini Mental State Examination (MMSE), Ascertain Dementia Eight-Item Informant Questionnaire (AD8), and Mini Nutritional Assessment (MNA) were used to assess the cognitive status and nutritional habits of this population.

Results

A total of 144 patients (mean age 75.3 years, 77.1% female) participated in this study. Forty percent of patients had AD8 and MMSE scores consistent with cognitive impairment and possible dementia. Age (p < 0.01), lower educational level (p < 0.01), history of stroke (p < 0.01), history of intracerebral hemorrhage (p < 0.01), diabetes mellitus (p < 0.01), and malnutrition (p < 0.01) were statistically significant risk factors for cognitive impairment. Exercise was found to be protective against cognitive decline in our study group (p < 0.03). Gender, ethnicity, location, head trauma, Parkinson disease, hypercholesterolemia, myocardial infarction, thyroid disease, depression, anxiety, and family history of dementia were not found to be associated with cognitive decline in this population.

Conclusions

The prevalence of cognitive impairment and possible dementia is 18–21% at age 65 and 54–60% at age 85 in Cumbayá, Quito, Ecuador. The major risk factors for cognitive impairment in this population are age, low educational level, malnutrition, prior stroke, prior intracerebral hemorrhage, and diabetes. Protective factors for cognitive decline include exercise and possibly modest consumption of alcohol.

## Introduction

Cognitive impairment and dementia are characterized by decline in intellectual functioning sufficient to interfere with activities of daily living. Alzheimer disease (AD) is the most common type of dementia in the elderly. It is associated with increased age and is an irreversible, degenerative disorder of the brain that progresses over several years. The course of the disease varies on a case-by-case basis. Approximately 10% of 65-year-olds suffer from AD, although most symptoms appear after this age, with the prevalence reaching 50% by 85 years of age. The neurodegeneration of AD leads to a gradual change in personality and behavior, impairment in cognition (thinking, decision-making, executive function, language), and eventually complete loss of intellectual function [1-4].

It has been reported that more than five million individuals in the United States of America currently suffer from this disease and that the prevalence doubles every five years. Estimates suggest that by the year 2050 approximately 13 million people will suffer from AD if population trends are maintained without development of a cure. On average, a person with AD will live between eight and 20 years following the onset of symptoms. The average life expectancy after diagnosis depends on the age of the person, stage of illness at diagnosis, and comorbid diseases. In the United States in 2017, the sum of direct and indirect costs of caring for patients with AD was estimated to have been $259 billion. Given its prevalence as well as its impact on individual patients, families, societies, and healthcare systems, AD and other dementing illnesses represent a global health issue warranting robust attention [5-8].

In Ecuador, dementia has not been extensively investigated. Our research group conducted a study in 2011 in the province of Pichincha, in which we showed that the prevalence of dementia in this region is likely higher than in developed countries. We also found that the main risk factors for cognitive impairment in this area of the world were increased age and low level of education [9,10]. The majority of studies about cognitive decline and dementia have been carried out abroad, and represent a population very different from that of Ecuador in terms of ethnicity, education, nutrition, and several other features. Our objectives in this study were to estimate the prevalence of cognitive impairment in Ecuadorian people over 65, search for additional risk factors, and validate our previous work.

## Materials and methods

This is a cross-sectional neuroepidemiological study carried out between September 2016 and May 2017. The subjects of the study were adults older than 65 living in Cumbayá, a parish of the metropolitan district of Quito in the province of Pichincha in Ecuador. The sample size of the study was calculated according to the Ecuadorian National Census of 2010, which determined that Cumbayá has a population of 31,463, of which 1,810 are over 65 years old.

To determine the size of the total sample *n*, the following parameters were taken into account: 95% confidence interval, 5% margin of error, estimated prevalence of dementia of 0.6, and estimated population size of patients over age 65 of 1,810. Using this information, along with Statistical Analysis Software (SAS), it was determined that a minimum sample of 82 subjects was needed. In this study, we were able to recruit 144 patients, which was well above the minimum needed. All the patients in this study gave informed consent to participate. The informed consent was approved by the Institutional Review Board of the Central University of Ecuador medical school.

The inclusion criteria were as follows: age greater than 65 and agreement by informed consent. The exclusion criteria included: age less than 65 and decline of offer to participate. The study subjects were selected randomly. The study units were the addresses of homes, shelters, and care centers for the elderly, which were randomly selected from different neighborhoods of Cumbayá (Lumbisí, San Juan, Pinsha, Rojas, Santa Inés, and Cumbayá Centro).

Data was collected in the form of questionnaires for each of the assessments: Mini Mental State Examination (MMSE), Ascertain Dementia Eight-Item Informant Questionnaire (AD8), and Mini Nutritional Assessment (MNA) [11-13]. Cognitive function based on MMSE scores is described as follows: >23 is considered normal cognition, 19–23 is mild cognitive impairment, 10–18 is moderate impairment, and <10 is severe impairment. On the AD8, a score of zero to one indicates normal cognition, while two or greater denotes cognitive impairment. The MNA categorizes patients scoring 12–14 points as having normal nutritional status, those with 8–11 points as being at risk for malnutrition, and those with fewer than seven points as malnourished. Additionally, MMSE testing was adjusted for education by considering only testable items in the score. For example, in the case of illiterate patients no points were taken from categories that required reading. Medical history and lifestyle habits were also recorded for each study subject. In calculating the prevalence of cognitive decline in the population based on MMSE scores, we considered “cognitive decline” to include those patients with either moderate or severe impairment. Mild cognitive impairment was not part of this study. The information was stored in Microsoft Excel for further organization and investigation.

In order to measure the association between the values obtained from the MMSE, MNA, and AD8 – classified according to reference values obtained from the literature – and social, demographic, and other risk factor variables, the Fisher exact test was used. The concordance between the classification of dementia obtained from the MMSE and AD8 instruments was measured by the kappa coefficient, with its respective 95% confidence interval.

## Results

**Figure 1:**
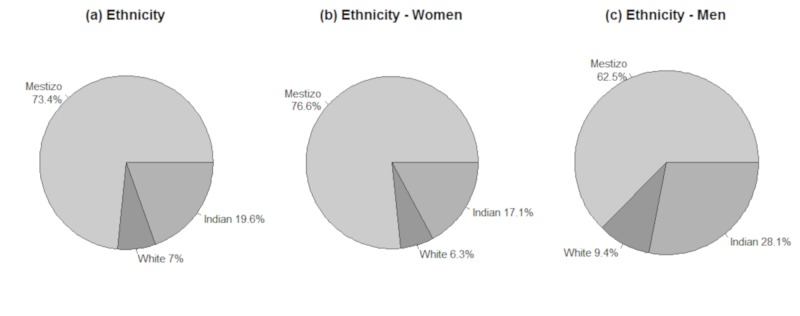
Participant ethnicity.

The sample consisted of 144 participants, with 111 (77.1%) women with a mean age of 75.3, and 33 (22.9%) men with a mean age of 76.1. The majority of participants were mestizos (73.4%), with the remainder classified as white (7.0%) or indigenous (19.6%) (Table 1). Figure 1 illustrates the ethnic composition of our sample population, both as a whole and by gender. This distribution is consistent with the data found in the last Ecuadorian census, in which 71.99% of the people were self-described as mestizos. In terms of education, 53.8% of participants had three to six years of education and 24.5% had no education. In the Santa Inés region, a greater proportion of the population had no education, while the regions of Rojas and Cumbayá Centro had the highest number of individuals with more than six years of education. Women as group had fewer years of education than men (Table 2).

**Table 1:** Participant demographics, ethnicity, and education by neighborhood/location.

		Location					
	Total	Lumbisí 1	*Lumbisí 2*	Rojas	San Juan Pinsha	Santa Inés	Cumbayá Centro
	n = 144	n = 48	n = 14	n = 19	n = 24	n = 11	n = 28
	n (%)	n (%)	n (%)	n (%)	n (%)	n (%)	n (%)
Gender							
Female	111 (77.1)	37 (77.1)	12 (85.7)	13 (68.4)	16 (66.7)	8 (72.7)	25 (89.3)
Male	33 (22.9)	11 (22.9)	2 (14.3)	6 (31.6)	8 (33.3)	3 (27.3)	3 (10.7)
Ethnicity							
Mestizo	105 (73.4)	26 (55.3)	9 (64.3)	18 (94.7)	19 (79.2)	10 (90.9)	23 (82.1)
White	10 (7.0)	7 (14.9)	0	1 (5.3)	1 (4.2)	1 (9.1)	0
Indian	28 (19.6)	14 (29.8)	5 (35.7)	0	4 (16.6)	0	5 (17.9)
Education							
No education	36 (24.5)	12 (25.5)	3 (21.4)	6 (31.6)	4 (16.7)	6 (54.5)	4 (14.3)
One to two years	20 (14.0)	3 (6.4)	4 (28.6)	0	6 (25.0)	1 (9.1)	6 (21.4)
Three to six years	77 (53.8)	31 (66.0)	6 (42.9)	10 (52.6)	13 (54.2)	4 (36.4)	13 (46.4)
> Six years	11 (7.7)	1 (2.1)	1 (7.1)	3 (15.8)	1 (4.1)	0	5 (17.9)

**Table 2:** Participant age, ethnicity, and education by gender.

	Gender					
	Women			Men		Fisher exact test
	n	%		n	%	
Age						0.74
65–69 years	21	19.3		7	21.2	
70–74 years	36	33		7	21.2	
75–79 years	23	21.1		8	24.2	
80–84 years	17	15.6		7	21.2	
>85 years	12	11		4	12.1	
Ethnicity						0.28
Mestizo	85	76.6		20	62.5	
White	7	6.3		3	9.4	
Indian	19	17.1		9	28.1	
Education years						0.03
No education	32	28.8		3	9.4	
One to two years	17	15.3		3	9.4	
Three to six years	56	50.5		21	65.6	
> Six years	6	5.4		5	15.6	

Multiple variables potentially associated with cognitive dysfunction and dementia were investigated. The most frequent conditions were hypertension (60.4%), depression (43.1%), anxiety (42%), hypercholesterolemia (29.2%), diabetes mellitus (25.7%), and traumatic brain injury (23.6%). In contrast, the least prevalent conditions included Parkinson disease (1.4%), myocardial infarction (0.7%), and tobacco use (2.1%). It should be noted that family history of dementia was found in only 2.8% of individuals from the various regions. Regarding healthy habits, it was observed that 28.5% of the individuals in our study population exercised and 79.9% reported routine walking.

The results of the MMSE showed that older age is a risk factor for cognitive decline. At age 65, 21.4% of people in our population showed evidence of cognitive decline, and for those 80 years of age or older this rose to 50% (p < 0.01). Poor education was also a significant risk factor for cognitive decline and dementia (p < 0.01). The group with no education was observed to contain a higher fraction of individuals with moderate and severe dementia, as compared with educated groups. Cerebral hemorrhage and cerebral infarction were additional risk factors associated with cognitive decline and dementia (p < 0.01). We found no statistically significant association between cognitive decline and the following variables: gender, geographic location, ethnicity, Parkinson disease, head trauma, hypertension, hypercholesterolemia, diabetes mellitus, thyroid disease, myocardial infarction, depression, anxiety, alcohol use, and smoking. Walking and other exercise were associated with decreased prevalence of cognitive decline. Interestingly, a family history of dementia was not associated with greater cognitive decline in this population (p = 0.52) (Table 3).

**Table 3:** Study variables and MMSE scores. MMSE: Mini Mental State Examination

	MMSE score											
	Normal			Mild			Moderate			Severe		Fisher exact test
	n	%		n	%		n	%		n	%	
Gender												0.68
Female	66	54.5		19	17.1		21	18.9		5	4.5	
Male	24	72.8		4	12.1		4	12.1		1	3	
Age												<0.01
65–69 years	22	78.6		2	7.1		4	14.3		0		
70–74 years	35	81.4		4	9.3		4	9.3		0		
75–79 years	18	58.1		4	12.9		8	25.8		1	3.2	
80–84 years	11	45.8		7	29.2		4	16.7		2	8.3	
>85 years	4	25		6	37.6		3	18.7		3	18.7	
Ethnicity												0.26
Mestizo	67	63.8		17	16.2		18	17.1		3	2.9	
White	4	40		1	10		4	40		1	10	
Indian	18	64.3		5	17.9		3	10.7		2	7.1	
Education years												<0.01
No education	10	28.6		6	17.1		14	40		5	14.3	
One to two years	16	80		2	10		2	10		0		
Three to six years	52	67.5		15	19.5		9	11.7		1	1.3	
> Six years	11	100		0			0			0		
Intracerebral hemorrhage												<0.01
Yes	3	33.3		0			6	66.7		0		
No	87	64.4		23	17		19	14.1		6	44.4	
Ischemic stroke												<0.01
Yes	2	13.3		5	33.3		6	40		2	13.3	
No	88	68.2		18	14		19	14.7		4	3.1	
Parkinson disease												0.61
Yes	1	50		0			1	50		0		
No	89	62.7		23	16.2		24	16.9		6	4.2	
Head trauma												0.1
Yes	16	47.1		7	20.6		8	23.5		3	8.8	
No	74	67.3		16	14.6		17	15.4		3	2.7	
Hypertension												0.43
Yes	50	57.5		15	17.2		17	19.5		5	5.8	
No	40	70.2		8	14		8	14		1	1.8	
Hypercholesterolemia												0.13
Yes	22	52.4		6	14.3		12	28.6		2	4.7	
No	68	66.7		17	16.7		13	12.7		4	3.9	
Diabetes mellitus												0.1
Yes	18	48.7		9	24.3		7	18.9		3	8.1	
No	72	67.3		14	13.1		18	16.8		3	2.8	
Thyroid disease												1
Yes	3	60		1	20		1	20		0		
No	87	62.6		22	15.8		24	17.3		6	4.3	
Myocardial infarction												1
Yes	1	100		0			0			0		
No	89	62		23	16.1		25	17.5		6	4.2	
Depression												0.34
Yes	38	61.3		7	11.3		14	22.6		3	4.8	
No	52	63.4		16	19.5		11	13.4		3	3.7	
Anxiety												0.78
Yes	40	66.7		10	16.7		8	13.3		2	3.3	
No	50	60.2		13	15.7		16	19.3		4	4.8	
Alcohol use												0.44
Yes	13	72.2		4	22.2		1	5.6		0		
No	77	61.1		19	15.1		24	19		6	4.8	
Smoking												0.15
Yes	1	33.3		2	66.7		0			0		
No	89	63.1		21	14.9		25	17.7		6	4.3	
Exercise												0.04
Yes	32	78		6	14.6		3	7.3		0		
No	58	56.3		17	16.5		22	21.4		6	5.8	
Walking												<0.01
Yes	74	64.4		22	19.1		16	13.9		3	2.6	
No	16	55.2		1	3.5		9	31		3	10.3	
Family history of dementia												0.52
Yes	2	50		1	25		1	25		0		
No	88	63.3		21	15.1		24	17.3		6	4.3	

The results of the AD8 showed that women in our sample had a higher prevalence of cognitive decline and dementia than men (p < 0.01). As in the case of the MMSE, the results of the AD8 showed that age and educational level have a relationship with cognitive decline and dementia. At age 65, 17.44% of individuals in our sample had abnormal results, and by 80 years, 60.9% demonstrated cognitive impairment (p < 0.01). According to the AD8 tests administered, the group with no education had the highest prevalence of cognitive decline and dementia, at 66.7% (Table 4).

**Table 4:** Study variables and AD8 scores. AD8: Ascertain Dementia Eight-Item Informant Questionnaire

	AD8 scores										
	0–1 point						≥2 points				Fisher exact test
	n		%				n		%		
Gender											<0.01
Female	42		47.2				47		52.8		
Male	22		84.6				4		15.4		
Age											0.03
65–69 years	19		82.6				4		17.4		
70–74 years	17		56.7				13		43.3		
75–79 years	13		50				13		50		
80–84 years	9		39.1				14		60.9		
>85 years	6		46.1				7		53.9		
Ethnicity											0.2
Mestizo	43		53.1				38		46.9		
White	4		44.4				5		55.6		
Indian	17		70.8				7		29.2		
Education years											0.06
No education	8		33.3				16		66.7		
One to two years	7		50				7		50		
Three to six years	43		64.2				24		35.8		
> Six years	6		66.7				3		33.3		
Intracerebral hemorrhage											0.46
Yes	3		37.5				5		62.5		
No	61		57				46		43		
Ischemic stroke											0.11
Yes	3		30				7		70		
No	61		58.1				44		41.9		
Parkinson disease											0.44
Yes	0						1		100		
No	64		56.1				50		43.9		
Head trauma											0.19
Yes	12		44.4				15		55.6		
No	52		59.1				36		40.9		
Hypertension											0.57
Yes	36		52.9				32		47.1		
No	28		59.6				19		40.4		
Hypercholesterolemia											0.29
Yes	14		46.7				16		53.3		
No	50		58.8				35		41.2		
Diabetes mellitus											<0.01
Yes	9		32.1				19		67.9		
No	55		63.2				32		36.8		
Thyroid disease											0.63
Yes	3		75				1		25		
No	61		55				50		45		
Myocardial infarction											0.44
Yes	0						1		100		
No	64		56.1				50		43.9		
Depression											0.57
Yes	26		52				24		48		
No	38		58.5				27		41.5		
Anxiety											0.57
Yes	24		52.2				22		47.8		
No	40		58.8				28		41.2		
Alcohol use											0.02
Yes	12		85.7				2		14.3		
No	52		51.5				49		48.5		
Smoking											1
Yes	1		50				1		50		
No	63		55.8				50		44.2		
Exercise											0.03
Yes	25		71.4				10		28.6		
No	39		48.8				41		51.2		
Walking											1
Yes	49		55.7				39		44.3		
No	15		55.6				12		44.4		
Family history of dementia											0.25
Yes	3		100				0				
No	61		54.5				51		45.5		

The AD8 found no statistically significant relationship between dementia and the following: gender, geographic location, ethnicity, Parkinson disease, head trauma, hypertension, hypercholesterolemia, thyroid disease, myocardial infarction, depression, anxiety, and smoking. The AD8 results revealed that diabetes mellitus was associated with cognitive decline and dementia (p < 0.01), which was not observed with the MMSE. However, alcohol consumption and exercise were associated with lower AD8 scores. Additionally, family history of dementia appeared to be a risk factor for cognitive decline and dementia when the AD8 was administered (Table 4).

We assessed the ability of the MMSE and AD8 to identify individuals with cognitive problems using two strategies. The first compared the two instruments based on their ability to identify abnormal results, and then calculated the correlation coefficient (kappa) between them. Table 5 shows the results of the first approach, indicating that the AD8 classified 55.6% of individuals as normal, and the MMSE classified 62.5% this way. The correlation coefficient revealed moderate agreement between the two instruments (kappa = 0.389).

**Table 5:** Comparison between the MMSE and AD8 in the detection of cognitive impairment. MMSE: Mini Mental State Examination; AD8: Ascertain Dementia Eight-Item Informant Questionnaire.

	Total	Normal		Abnormal	
	n	n	%	n	%
MMSE score	144	90	62.5	54	37.5
AD8 score	115	64	55.6	51	44.4

The MNA found that malnutrition correlated with following: female gender, intracerebral hemorrhage, Parkinson disease, hypertension, thyroid disease, alcohol use, and depression (Table 6). The MNA results showed no statistically significant association between poor nutritional status and the following variables: age, geographical location, education, ethnicity, ischemic stroke, head trauma, myocardial infarction, smoking, and anxiety. Individuals who consume alcohol tended to have better nutritional status.

**Table 6:** Study variables and the MNA. MNA: Mini Nutritional Assessment.

	Nutritional status																
	Good						Nutritional Risk						Malnourished				Fisher exact test
	n		%				n		%				n		%		
Gender																	<0.01
Female	2		1.9				93		86.1				13		12		
Male	8		24.2				22		66.7				3		9.1		
Age groups																	0.6
65–69 years	0						22		88				3		12		
70–74 years	2		4.6				37		86.1				4		9.3		
75–79 years	5		16.1				22		71				4		12.9		
80–84 years	2		8.3				20		83.3				2		8.3		
>85 years	1		6.2				13		81.3				2		12.5		
Ethnicity																	0.66
Mestizo	6		5.9				85		83.3				11		10.8		
White	0		0				8		80				2		20		
India	3		10.7				22		78.6				3		10.7		
Education years																	0.62
No education	1		2.9				27		79.4				6		17.7		
One to two years	1		5.2				17		89.5				1		5.3		
Three to six years	7		9.2				60		79				9		11.8		
> Six years	0						11		100				0				
Intracerebral hemorrhage																	0.01
Yes	2		22.2				4		44.4				3		33.3		
No	8		6.1				111		84.1				13		9.8		
Ischemic stroke																	0.66
Yes	0						14		93.3				1		6.7		
No	10		7.9				101		80.2				15		11.9		
Parkinson disease																	0.02
Yes	0						0						2		100		
No	10		7.2				115		82.7				14		10.1		
Head trauma																	0.35
Yes	1		2.9				31		91.2				2		5.9		
No	9		8.4				84		78.5				14		13.1		
Hypertension																	0.02
Yes	2		2.3				75		87.2				9		10.5		
No	8		14.6				40		72.3				7		12.7		
Hypercholesterolemia																	0.43
Yes	1		2.4				36		85.7				5		11.9		
No	9		9.1				79		79.8				11		11.1		
Diabetes mellitus																	0.14
Yes	2		5.6				33		91.7				1		2.8		
No	8		7.6				82		78.1				15		14.3		
Thyroid disease																	0.01
Yes	0						2		40				3		60		
No	10		7.3				113		83.1				13		9.6		
Myocardial infarction																	1
Yes	0						1		100				0				
No	10		7.1				114		81.4				16		11.4		
Depression																	<0.01
Yes	4		6.4				44		71				14		22.6		
No	6		7.6				71		89.9				2		2.5		
Anxiety																	0.28
Yes	3		5.1				47		79.7				9		15.2		
No	7		8.6				68		84				6		7.4		
Alcohol use																	0.02
Yes	4		22.2				14		77.8				0				
No	6		4.9				101		82.1				16		13		
Smoking																	0.46
Yes	0						2		66.7				1		33.3		
No	10		7.2				113		81.9				15		10.9		
Exercise																	0.52
Yes	4		9.8				34		82.9				3		7.3		
No	6		6				81		81				13		13		
Walking																	0.12
Yes	8		7				96		84.2				10		8.8		
No	2		7.4				19		70.4				6		22.2		
Family history of dementia																	0.15
Yes	1		25				2		50				1		25		
No	9		6.6				113		83.1				14		10.3		

As part of our analysis, nutritional status was compared with cognitive function as measured by the MMSE and AD8. A good nutritional status was associated with normal MMSE scores. MMSE scores were normal in 100% of patients with good nutritional status, 62.6% of patients at nutritional risk, and 37.5% of malnourished patients. The AD8 results correlated with this, showing normal results in 80%, 54.3%, and 44.0% in these groups, respectively (Tables 7, 8).

**Table 7:** Nutritional status and MMSE scores. MMSE: Mini Mental State Examination

	MMSE score								
	Normal		Mild		Moderate		Severe		Fisher exact test
	n	%	n	%	n	%	n	%	
Nutritional status									0.05
Good	10	100	0		0		0		
Nutritional risk	72	62.6	20	17.4	19	16.5	4	3.5	
Malnutrition	6	37.5	3	18.8	5	31.2	2	12.5	

**Table 8:** Nutritional status and AD8 scores. AD8: Ascertain Dementia Eight-Item Informant Questionnaire

	AD8 score				
	0–1 point		≥2 points		Fisher exact test
	n	%	n	%	
Nutritional status					0.25
Good	8	80	2	20	
Nutritional risk	51	54.3	43	45.7	
Malnutrition	4	44	5	55.6	

## Discussion

The overall prevalence of cognitive impairment in adults over age 65 in Cumbayá, Ecuador was estimated at 37.5% by the MMSE and 44.4% by the AD8. These data are consistent with those of our previous study in Ecuador and studies done in other parts of the world [10]. The results of this study suggest that both the AD8 and MMSE are valid instruments for identifying cognitive impairment in this population. Additionally, MMSE scores correlated consistently with AD8 scores. Thus, both exams appear to be reliable tools in assessing cognitive function in this study group. To our knowledge this is the first time that MMSE has been used and validated in a clinical study in the Ecuadorian population.

In our study, age was observed to be a statistically significant risk factor for cognitive decline and dementia (p < 0.01). The MMSE scores demonstrated cognitive impairment in 22.4% in the 65-year-old population and 54.2% in the 80-year-old population, and the AD8 results showed similar prevalence in each group, at 18.4% and 60.9%, respectively. These values are somewhat higher than those reported in the international literature, in which the prevalence of dementia has been reported at 11% at age 65 and 50% at age 85 [4]. One possible explanation for this is that our instruments are used to detect cognitive impairment and not to diagnose dementia, which requires more formal physical and neurological examination, imaging studies, and laboratory testing. Another reason for the higher prevalence observed in our study population may also be the presence of other neurological or psychiatric illnesses in our patients that resemble dementia. For example, several reversible causes of dementia such as vitamin B12 deficiency, hypothyroidism, tertiary syphilis, autoimmune diseases, and pseudodementia, defined as cognitive impairment as a symptom of depression, may have been present but undiagnosed in some individuals whose testing showed evidence of cognitive decline. Despite this, our data are valid and illustrate the widely observed correlation between increased age and risk of cognitive impairment. The AD8 appears to be more sensitive in the detection of cognitive decline and dementia than the MMSE, and the results obtained with the AD8 are consistent with those of previous studies [9,10].

The results of this study suggest that a low level of education is a major risk factor for cognitive impairment and dementia (p < 0.01) in this population. In Cumbayá, most residents had an education under six years in length (92.3%). 24.5% of these patients had no education, 14% had one to two years, and 53% had three to six years of education. The women in our study group tended to have much less education than the men. Thirty-two percent of females had no education (zero years of study) compared to 3% of men, which may reflect a generational and cultural phenomenon in which men are more encouraged to pursue an education than women. This is an important finding since poor education is a well-recognized and modifiable risk factor for dementia worldwide [14].

History of cerebral infarction (p < 0.01) and intracerebral hemorrhage (p < 0.01) are statistically significant risk factors for cognitive decline in this sample, a valid and relevant finding in our study, since cerebrovascular events are responsible for vascular dementia, the second most common variant of dementia after AD. Cerebral infarction and intracerebral hemorrhage can cause cognitive impairment due to damage to specific areas in the brain such as the cerebral lobes, mainly the temporal and parietal lobes, as well as brain nuclei such as the thalamus, hippocampus, and basal ganglia. Damage to these structures results in sudden and permanent cognitive impairment. This finding is important because the main risk factors for cerebrovascular disease are hypertension, hyperlipidemia, and diabetes mellitus, all of which are common diseases that are treatable once identified.

The results of the AD8 suggest that diabetes mellitus (p < 0.01) is a statistically significant risk factor for cognitive impairment and dementia. As described above, diabetes increases the risk for vascular causes of dementia, such as cerebral infarction or hemorrhage, and independently increases the risk for cognitive decline. This result is consistent with findings in the international literature and is meaningful because diabetes is also a modifiable risk factor and appropriate treatment may thwart the risk of developing dementia later in life. The AD8 results also propose that physical activity (p < 0.03) is a statistically significant protective factor against cognitive impairment and dementia. In other words, patients with a normal AD8 who exercise have been shown to have normal cognition by other testing. This has been validated by previous studies, which show that exercise decreases the risk for Alzheimer disease.

In the literature, family history of dementia is another well-known risk factor for cognitive impairment. In our study, family history of dementia was reported in only 2.8% of the population and was not found to be a statistically significant risk factor for cognitive decline (p = 0.52). This may be explained by the possibility that our cohort of individuals age 65 or greater represent an epidemiological transition in Ecuador. In other words, this may be the first group in this region to have a life expectancy in an age range where neurocognitive decline generally manifests, so prior to this, much of the population likely passed away before the onset of dementia so it would not have been diagnosed in them. This epidemiological phenomenon is important because it now introduces the possibility of reaching an age at which cognitive impairment becomes prevalent, necessitating interventions to identify and prevent its development [2,3].

Ethnicity was not a significant risk factor for cognitive dysfunction and dementia in our sample population (p = 0.26). In contrast, in the United States, ethnic minority groups such as African Americans, Latinos, and Caribbean Americans have been shown to have higher prevalence of dementia than Caucasians [15-17]. Possible explanations for the lack of observed difference in risk between ethnicities in our study include the relatively small sample size of our study, and the fact that the diagnosis of dementia requires more formal neurocognitive testing.

The AD8, but not MMSE, scores showed that subjects who consume alcohol (p < 0.02) had a lower risk of cognitive impairment and dementia. These data should be considered with caution, since alcohol consumption in excess is a known cause of cognitive impairment and dementia. The association found in our study has been reported in other populations in which consumption of modest amounts of alcohol and a Mediterranean diet have been identified as preventive factors for dementia [18-19].

The AD8 and MMSE showed a moderate correlation with one another in terms of detecting cognitive decline (kappa = 0.389). This correlation is significant since both instruments are used for the evaluation of cognitive deterioration and dementia, although the source of information differs between the two tests. The AD8 is subjective, based on the interpretation of a relative who knows the patient, while the MMSE is more objective since the questions are posed to the individual being evaluated. Using both rather than one instrument may be beneficial, especially given certain confounders, such as low educational level in the MMSE. The AD8, on the other hand, allows evaluation of the individual through an informant regardless of education. In either case, administering both may be preferred, and this is especially true of our study population given the prevalence of poor education.

We found an association between nutritional risk and malnutrition as classified by the MNA and the following: Parkinson disease, hypertension, thyroid disease, depression, and alcohol use (Table 6). To the best of our knowledge, this study represents the first use of the MNA for research purposes in Ecuador in people with suspected cognitive impairment. This instrument has been validated in many languages ​​and was easy to administer in our study patients. We found that in people over 65 years of age, 81% are at nutritional risk and 11.3% are malnourished. These data are similar to those of international reports using the same instrument. For example, studies done in the United Kingdom estimate that the prevalence of malnutrition is 16% in patients over age 65. The similarity of our data to that of other countries gives validity to our study.

When individual scores on the MNA were compared with those on the AD8 and MMSE, we observed that good nutritional status was associated with normal cognition. On the other hand, malnutrition appeared to be associated with cognitive impairment and dementia (p = 0.05) (Tables 7, 8). These findings are consistent with reports in the literature, which show that malnutrition is associated with increased risk for dementia and Alzheimer disease [20-21].

These results should be considered in the context of study limitations, namely that patients identified as having cognitive impairment have not undergone formal medical and neurological examination. However, identification of these individuals who screen positive for cognitive impairment is significant because it raises awareness of such issues and may prompt confirmatory testing and appropriate treatment if needed.

Based on our observations in this study, greater recognition of cognitive impairment is needed in this population with increasing life expectancy and many relevant risk factors. There is a need for policies and systems that increase access to diagnostic, treatment, and preventive services for those with cognitive decline and dementia in Ecuador.

## Conclusions

The prevalence of cognitive impairment in Cumbayá, Quito, Ecuador is 18–22% at age 65 and 54–60% at age 80. This result depicts the current state of mental and cognitive health in the elderly population of Ecuador, especially when compared with data from similar studies conducted in developed countries, where the prevalence of dementia is often lower. The main risk factors for cognitive decline and dementia identified in this study population are increased age, lack of education, ischemic stroke, intracerebral hemorrhage, diabetes, and malnutrition. The main protective factors for cognitive impairment in this population include good nutrition and physical exercise.

Based on these findings, opportunities to reduce the incidence of cognitive decline and Alzheimer disease in the aging population of Ecuador should be sought out, beginning with addressing modifiable risk factors. This may include increasing access to education, decreasing risk for cerebrovascular events by diagnosing and managing diseases such as hypertension, hyperlipidemia, and diabetes, and encouraging healthy diet and physical activity. This study shows that the MMSE and AD8 screening tests are effective in detecting cognitive impairment and possible dementia in Ecuadorian patients, and that the MNA is a valid tool for nutritional assessment in this population.
